# The Influence of the Stimulus Design on the Harmonic Components of the Steady-State Visual Evoked Potential

**DOI:** 10.3389/fnhum.2020.00343

**Published:** 2020-09-09

**Authors:** Benjamin Solf, Stefan Schramm, Maren-Christina Blum, Sascha Klee

**Affiliations:** Institute for Biomedical Engineering and Informatics, Technische Universität Ilmenau, Ilmenau, Germany

**Keywords:** steady-state visual evoked potentials, ssVEP, flicker stimulation, harmonic components, stimulus eccentricity, ocular stray light

## Abstract

Steady-state visual evoked potentials (ssVEPs) are commonly used for functional objective diagnostics. In general, the main response at the stimulation frequency is used. However, some studies reported the main response at the second harmonic of the stimulation frequency. The aim of our study was to analyze the influence of the stimulus design on the harmonic components of ssVEPs. We studied 22 subjects (8 males, mean age ± SD = 27 ± 4.8 years) using a circular layout (*r*_1_ = 0–1.6°, *r*_2_ = 1.6–3.5°, *r*_3_ = 3.5–6.4°, *r*_4_ = 6.4–10.9°, and *r*_5_ = 10.9–18°). At a given eccentricity, the stimulus was presented according to a 7.5 Hz square wave with 50% duty cycle. To analyze the influence of the stimulus eccentricity, a background luminance of 30 cd/m^2^ was added to suppress foveal stray light effects; to analyze the influence of simultaneous foveal and peripheral stimulations, stimulations are performed without stray light suppression. For statistical analysis, medians **M** of the amplitude ratios for amplitudes at the second harmonic to the first harmonic and the probability of the occurrence of the main response at the second harmonic **P(MCSH)** are calculated. For stimulations with foveal stray light suppression, the medians were **M**_**0**–**1.6**°_ = 0.45, **M**_**1.6**–**3.5**°_ = 0.45, **M**_**3.5**–**6.4**°_ = 0.76, **M**_**6.4**–**10.9**°_ = 0.72, and **M**_**10.9**–**18**°_ = 0.48, and the probabilities were **P**_**0–1.6**°_**(MCSH)** = 0.05, **P**_**1.6**–**3.5**°_**(MCSH)** = 0.05, **P**_**3.5**–**6.4**°_**(MCSH)** = 0.32, **P**_**6.4**–**10.9**°_**(MCSH)** = 0.29, and **P**_**10.9**–**18**°_**(MCSH)** = 0.30. For stimulations without foveal stray light suppression, the medians **M** were **M**_**0**–**1.6**°_ = 0.29, **M**_**1.6**–**3.5**°_ = 0.37, **M**_**3.5**–**6.4**°_ = 0.98, **M**_**6.4**–**10.9**°_ = 1.08, and **M**_**10.9**–**18**°_ = 1.24, and the probabilities were **P**_**0–1.6**°_**(MCSH)** = 0.09, **P**_**1.6**–**3.5**°_**(MCSH)** = 0.05, **P**_**3.5**–**6.4**°_**(MCSH)** = 0.50, **P**_**6.4**–**10.9**°_**(MCSH)** = 0.55, and **P**_**10.9**–**18**°_**(MCSH)** = 0.55. In conclusion, the stimulus design has an influence on the harmonic components of ssVEPs. An increase in stimulation eccentricity during extrafoveal stimulation leads to a transition of the main response to the second harmonic. The effect is enhanced by a simultaneous foveal stimulation.

## Introduction

In clinical practice, visual evoked potentials (VEPs) are commonly used for ophthalmologic diagnostics. A distinction is made between transient VEPs, as a response to a single stimulus event, and steady-state VEPs (ssVEPs), as a response to intermittent stimulation with short stimulus interval time (Regan, 1966). Due to the advantages of short recording time with a high number of responses, ssVEPs are used for objective functional diagnostics, e.g., in the determination of visual acuity (Bach et al., 2008) or contrast threshold (Norcia et al., 1989). Therefore, visual stimuli are presented to the subject, and one stimulation parameter, e.g., the stimulation contrast, is swept over the stimulation time (Regan, 1973; Tyler et al., 1979; Norcia and Tyler, 1985). The recorded signals are transformed into the frequency domain and amplitude and phase information are used (Bach and Meigen, 1999). A review of the different sweep VEP techniques and use cases is given in Almoqbel et al. (2008).

Due to the stimulation with intermittent stimuli, the evoked potential contains frequency components related to the stimulation frequency and its harmonics (Herrmann, 2001). In general, the evoked amplitude at the stimulation frequency (first harmonic) is used for functional diagnostics (Heinrich, 2010). However, some studies reported the main response (highest amplitude value in the frequency domain) at the second harmonic. In the VEP-based acuity estimation using pattern-onset stimulations, the main response occurs occasionally at the second harmonic (Heinrich et al., 2016). Another example is the electrophysiological determination of the stray light perception (Solf et al., 2019b). Therefore, the compensation of a veiling luminance induced by a peripheral stray light source in a foveal imaged test field is used. In the study by Solf et al. (2019b), 5 out of 10 subjects showed the main response at the second harmonic. A possible explanation is the stimulus design consisting of peripheral and foveal stimulation.

The stimulus frequency affects the shape of the responses to single stimuli. If the responses to single stimuli overlap, signal components of the single responses may remain. Thus, the resulting signal of overlapped responses may contain signal components that differ from the stimulus frequency, leading to higher harmonics (Heinrich, 2010). An entrainment of neuronal oscillations (Notbohm et al., 2016; Salchow et al., 2016) as well as resonance effect in the range of the higher harmonic components of the stimulation frequency (Herrmann, 2001) could lead to an increase of the higher harmonic signal components. These effects are probably due to the non-linear behavior of neural oscillators (Norcia et al., 2015; Labecki et al., 2016).

The effect of the stimulation parameters on the harmonic components of ssVEPs was analyzed in some studies. For example, Johansson and Jakobsson (2000) found significant higher amplitudes at high temporal frequencies in normal subjects than in stereo-blind subjects. Gulbinaite et al. (2019) analyzed the effect of attention on the amplitudes of the harmonic components for stimulation frequencies within the range of 3–80 Hz and found an opposite effect of attention on the individual resonance frequencies in the alpha and gamma band.

The aim of our study was to analyze the influence of the stimulus design on the higher harmonic components of ssVEPs using the stimulus design of the electrophysiological stray light measurement. Therefore, the occurrence of the main response at the first or second harmonic is of particular interest. We want to investigate the impact of latency differences caused by eccentricity of the stimulus on the occurrence of the main response at the first or second harmonic. Furthermore, we consider the impact of the combination of foveal and peripheral stimulation resulting from the stimulus design.

## Materials and Methods

### Subjects

A total of 22 healthy subjects (8 males, 14 females, mean age ± SD = 27 ± 4.8 years) with normal or corrected to normal vision participated in the study. The subjects have given written informed consent to publish these case details. The study was approved by the Ethics Committee of the Faculty of Medicine of the Friedrich Schiller University Jena and was conducted in compliance with the Declaration of Helsinki.

### Stimulation

**FIGURE 1:**
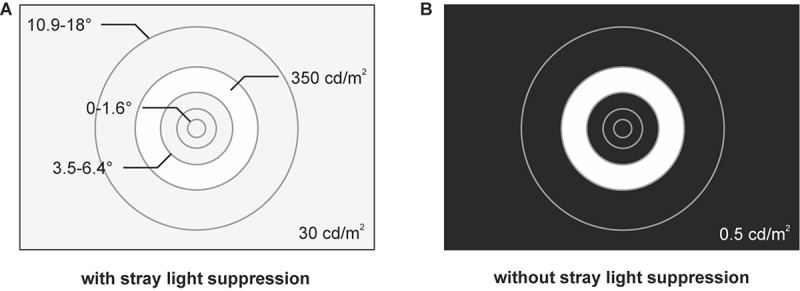
Stimuli for the two conditions of stray light suppression. **(A)** Stimulus design with a circular layout consisting of one circular stimulus (*r*_1_ = 0–1.6°) and four ring-shaped stimuli (*r*_2_ = 1.6–3.5°, *r*_3_ = 3.5–6.4°, *r*_4_ = 6.4–10.9°, and *r*_5_ = 10.9–18°). A background luminance of 30 cd/m^2^ is added to suppress foveal stray light effects, caused by the ring-shaped stimuli. **(B)** Stimulus design with the circular layout without foveal stray light suppression. The display had a background luminance of 0.5 cd/m^2^.

Stimulations were performed using a circular layout up to a radius of 18°, consisting of one circular stimulus and four ring-shaped stimuli. The dimensions of the stimuli were scaled according to the cortical magnification to activate nearly equivalent cortical areas in V1 (Levi and Klein, 1985; Horton and Hoyt, 1991; Baseler et al., 1994) resulting in the five stimulus eccentricities with the radii *r*_1_ = 0–1.6°, *r*_2_ = 1.6–3.5°, *r*_3_ = 3.5–6.4°, *r*_4_ = 6.4–10.9°, and *r*_5_ = 10.9–18° (Figure 1A).

**FIGURE 2:**
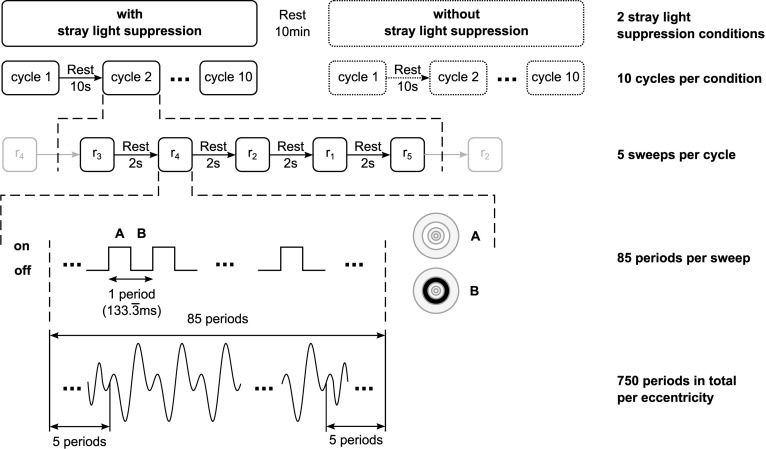
Schematic of the temporal structure of the stimulation. For each of the two stray light suppression conditions, 10 cycles per condition were performed. The stimuli were respectively presented for one sweep of 85 stimulus periods for each of the five eccentricities (*r*_1_ = 0–1.6°; *r*_2_ = 1.6–3.5°; *r*_3_ = 3.5–6.4°; *r*_4_ = 6.4–10.9°; *r*_5_ = 10.9–18°) in random order. The first and the last five periods of each sweep were excluded to reduce the engagement phase, resulting in 750 periods in total per eccentricity.

The stimuli had a luminance of 350 cd/m^2^ and a contrast of 99% (Michelson Contrast). A full-array LED-backlight liquid crystal display (LE-52F9BD, Samsung Corp., Seoul, South Korea) was used for the stimulation to ensure a homogenous illumination of the stimuli. The stimulation frequency was set to 7.5 Hz regarding the refresh rate of the display. The stimuli were respectively presented for one sweep of 85 stimulus periods for each of the five eccentricities in random order. The first and the last five periods of each sweep were excluded to reduce the engagement phase (Salchow et al., 2016). This was repeated 10 times (in total 50 sweeps) resulting in 750 periods per eccentricity (Figure 2). Binocular stimulations were performed for the following two different conditions of stray light suppression.

### Foveal Stray Light Suppression

Due to the bright ring-shaped stimuli, stray light, defined as a resulting equivalent veiling luminance (Cobb, 1911; Holladay, 1926; Stiles, 1929; van den Berg, 1995), has an impact on the evoked responses (Solf et al., 2019a). According to the young and healthy subjects and with respect to the applied stimulus dimensions, the resulting equivalent veiling luminance should be in a range of 2–3 cd/m^2^ (van den Berg, 1995). To suppress the foveal stray light effects, we added a background luminance of 30 cd/m^2^ (Figure 1A). Hence, the stimulation contrast of the foveal stimulation by equivalent veiling luminance is in the order of <5%.

### Without Foveal Stray Light Suppression

Without stimulation, the used display had a luminance of 0.5 cd/m^2^ (Figure 1B). Therefore, the equivalent veiling luminance of 2–3 cd/m^2^ caused by the ring-shaped stimuli resulted in a foveal stimulation contrast of about 60–70%. Thus, the equivalent veiling luminance had a measurable impact on the recorded ssVEPs.

### VEP Recording and Preprocessing

VEPs were recorded using Ag/AgCl ring electrodes with an electrode cap (EasyCap, *EASYCAP GmbH, Herrsching, Germany*) and an amplifier system (THERA PRAX, *neuroCare Group GmbH, Munich, Germany*) at a sample rate of 1024 Hz. The active electrode was placed at Oz, reference electrode at Fz, and ground at AFz according to the standard of the International Society for Clinical Electrophysiology of Vision (Odom et al., 2016). To synchronize the stimulation with the recorded data, we used a PIN photodiode placed in front of the stimulator, connected to the amplifier system. The recorded data were digitally bandpass filtered in forward and backward directions to avoid phase shifts with an infinite impulse response (IIR) Butterworth filter, with cut-off frequencies at 1 and 35 Hz to remove electrode drifts and signal distortions. The 10 sweeps for each eccentricity were separated and averaged using a rectangular window with a length of 2048 samples with no overlap. The averaged sweeps were transformed into the frequency domain using Fourier Transform (rectangular window) (Bach and Meigen, 1999).

### Data Analysis and Statistics

The signal analysis was performed using MATLAB V9.6 (*MathWorks Inc., Natick, United States*). The amplitude ratio of the amplitudes at the second harmonic to the first harmonic was calculated. The significance of the amplitudes at the first and second harmonic was determined. The two neighboring amplitudes in the frequency domain were used as noise estimation. The significance level was set to α = 0.05 (Meigen and Bach, 1999). Due to multiple testing, the significance level was adjusted to a significance value of *p*^∗^ = 0.025, corresponding to a SNR of 3.50. The amplitude ratio was calculated only if the amplitudes were significant at the first and/or the second harmonic. Responses with no significant amplitude (*p*^∗^ > 0.025) at the first or second harmonic were not analyzed. Ratios larger than 1 indicate a transition of the main component from the first harmonic to the second harmonic.

For statistical analysis, the distribution of the amplitude ratios is analyzed using boxplots. To evaluate the transition of the main response, the probability of the occurrence of the main response at the second harmonic **P(MCSH)** is calculated according to the sample size of the significant responses.

## Results

**FIGURE 3:**
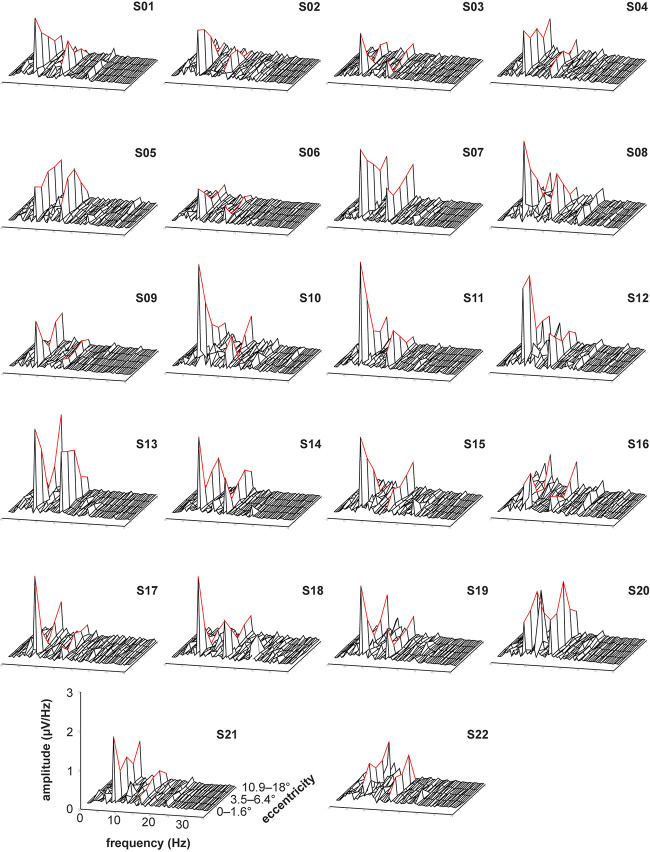
Recorded VEPs of the 22 subjects for the stimulations with foveal stray light suppression. The amplitude spectra after Fourier Transform are plotted from 0 to 35 Hz for the five stimulus eccentricities (*r*_1_ = 0–1.6°; *r*_2_ = 1.6–3.5°; *r*_3_ = 3.5–6.4°; *r*_4_ = 6.4–10.9°; *r*_5_ = 10.9–18°). The curve progression of the amplitudes at the first and second harmonics for the five eccentricities is highlighted in red. Most subjects show pronounced responses for the first and second harmonics of the stimulation frequency. The third harmonic is also partly pronounced but is not considered for further analysis. Additionally, the subjects S08, S10, S12, and S20 show alpha activity (at about 10 Hz).

**FIGURE 4:**
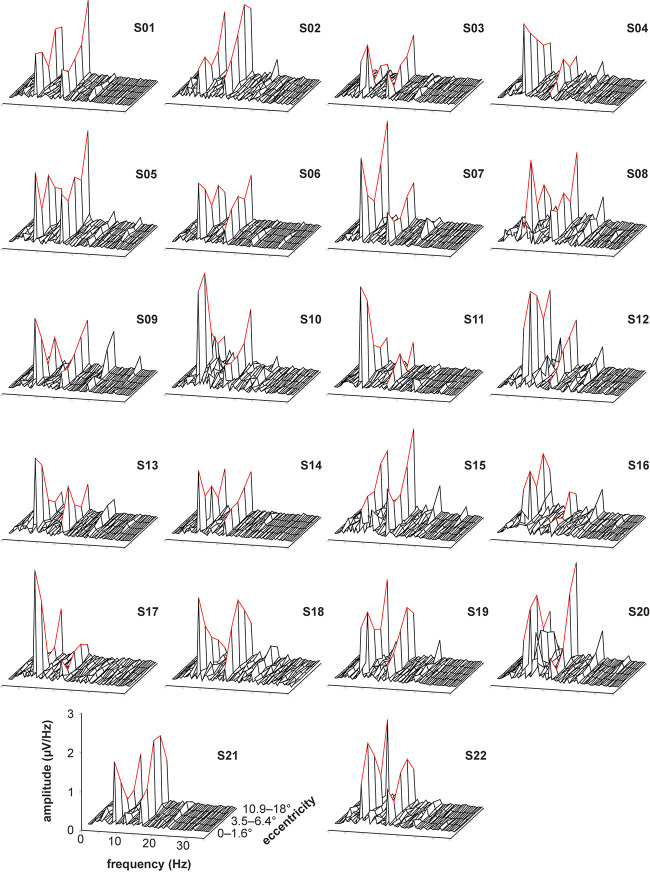
Recorded VEPs of the 22 subjects for the stimulations without foveal stray light suppression. The amplitude spectra after Fourier Transform are plotted from 0 to 35 Hz for the five stimulus eccentricities (*r*_1_ = 0–1.6°; *r*_2_ = 1.6–3.5°; *r*_3_ = 3.5–6.4°; *r*_4_ = 6.4–10.9°; *r*_5_ = 10.9–18°). The curve progression of the amplitudes at the first and second harmonics for the five eccentricities is highlighted in red. Most subjects show pronounced responses for the first and second harmonics of the stimulation frequency. The third harmonic is also partly pronounced but is not considered for further analysis. Additionally, the subjects S10, S12, and S20 show alpha activity (at about 10 Hz).

**FIGURE 5:**
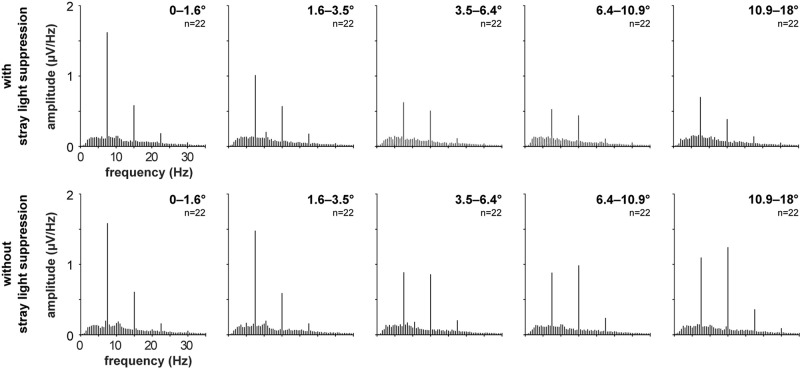
Grand mean spectra of the recorded VEPs of the 22 subjects for the stimulations with foveal stray light suppression and without foveal stray light suppression for the five stimulus eccentricities (*r*_1_ = 0–1.6°; *r*_2_ = 1.6–3.5°; *r*_3_ = 3.5–6.4°; *r*_4_ = 6.4–10.9°; *r*_5_ = 10.9–18°).

The recorded VEPs of the 22 subjects are shown in Figure 3 for the stimulations with foveal stray light suppression and in Figure 4 for the stimulations without foveal stray light suppression. In each subplot, the amplitude spectra after Fourier Transform are plotted from 0 to 35 Hz for the five stimulus eccentricities (*r*_1_ = 0–1.6°; *r*_2_ = 1.6–3.5°; *r*_3_ = 3.5–6.4°; *r*_4_ = 6.4–10.9°; *r*_5_ = 10.9–18°). The curve progression of the amplitudes at the first and second harmonics for the five eccentricities is highlighted in red. The grand mean spectra of the recorded VEPs are shown in Figure 5.

Most subjects show pronounced amplitudes at the first and second harmonics of the stimulation frequency for the stimulations with (Figure 3) and without foveal stray light suppression (Figure 4). The third harmonic is partly pronounced but is not considered for further analysis. The number of subjects with significant amplitudes at the first harmonic (7.5 Hz), second harmonic (15 Hz) and at first or second harmonic for the two conditions of stray light suppressions are shown in Table 1. The summary of the *p*-values of all subjects for the amplitude at the first and second harmonics is shown in Supplementary Tables 1, 2.

**TABLE 1:** Number of subjects with significant amplitudes at the first harmonic (7.5 Hz), second harmonic (15 Hz), and at first or second harmonic for stimulations with and without foveal stray light suppression.

	Number of subjects with significant amplitudes					
	
	With stray light suppression			Without stray light suppression		
		
Eccentricity	7.5 Hz	15 Hz	7.5 Hz/15 Hz	7.5 Hz	15 Hz	7.5 Hz/15 Hz
0–1.6°	21	18	21	19	21	22
1.6–3.5°	21	17	22	22	19	22
3.5–6.4°	13	18	19	17	21	22
6.4–10.9°	15	16	17	20	21	22
10.9–18°	15	17	20	19	22	22

The amplitude ratios of the amplitude at the first and second harmonics are shown in Table 2 (with foveal stray light suppression) and in Table 3 (without foveal stray light suppression). Ratios >1 indicate a main response at the second harmonic. For stimulations with foveal stray light suppression, respectively, one subject showed the main response at the second harmonic for stimulations for stimulus eccentricity of *r*_1_ = 0–1.6° and *r*_2_ = 1.6–3.5°. For *r*_3_ = 3.5–6.4°, six subjects showed the main response at the second harmonic. Five subjects for *r*_4_ = 6.4–10.9° and six subjects for *r*_5_ = 10.9–18° showed the main response at the second harmonic. No amplitude ratios could be calculated for subject S22 for the stimulus eccentricity *r*_1_ = 0–1.6°; for subjects S06, S16, and S18 for *r*_3_ = 3.5–6.4°; for subjects S02, S06, S08, S10, and S17 for *r*_4_ = 6.4–10.9°; and for subjects S02 and S06 for *r*_5_ = 10.9–18° due to non-significant responses at the first and second harmonics.

**TABLE 2:** Amplitude ratios for stimulations with foveal stray light suppression.

	Amplitude ratio				
	
Subject	0–1.6°	1.6–3.5°	3.5–6.4°	6.4–10.9°	10.9–18°
S01	0.23	0.84	0.51	0.67	0.15
S02	0.18	0.45	0.61	–	–
S03	0.45	0.10	**1.36**	0.72	**1.03**
S04	0.22	0.45	0.44	0.33	0.44
S05	0.52	**1.38**	**1.10**	0.64	0.22
S06	0.53	0.21	–	–	–
S07	0.50	0.41	0.55	0.90	0.84
S08	0.25	0.95	0.89	–	0.48
S09	0.23	0.28	0.76	0.39	0.21
S10	0.17	0.46	0.00	–	**1.42**
S11	0.16	0.53	0.86	0.51	0.33
S12	0.45	0.30	0.62	0.70	0.47
S13	0.75	0.99	**4.47**	0.71	0.19
S14	0.50	0.57	0.68	0.73	**2.21**
S15	0.09	0.41	0.53	**4.34**	**8.38**
S16	0.63	0.38	–	**2.66**	0.84
S17	0.18	0.23	**3.55**	–	0.36
S18	0.49	0.97	–	**1.73**	**1.52**
S19	0.45	0.30	**1.04**	0.65	0.50
S20	**1.10**	0.96	**1.22**	**1.18**	**1.69**
S21	0.15	0.40	0.47	0.78	0.23
S22	–	0.63	0.79	**1.30**	0.03

**Amplitude ratios >1 indicate the main response at the second harmonic and are highlighted.**

**TABLE 3:** Amplitude ratios for stimulations without foveal stray light suppression.

	Amplitude ratio				
	
Subject	0–1.6°	1.6–3.5°	3.5–6.4°	6.4–10.9°	10.9–18°
S01	0.69	0.56	**1.49**	0.73	**1.60**
S02	0.74	0.98	**2.54**	**2.63**	**1.08**
S03	0.80	0.17	**3.82**	**1.75**	**3.94**
S04	0.11	0.23	0.62	0.45	0.63
S05	0.72	**1.34**	**1.00**	**1.24**	**2.92**
S06	0.27	0.62	**1.42**	0.70	**1.69**
S07	0.39	0.43	0.51	0.50	0.39
S08	**2.25**	0.36	**1.55**	0.62	**3.24**
S09	0.27	0.36	**1.98**	**1.08**	**7.96**
S10	0.26	0.20	0.52	**1.09**	**2.01**
S11	0.06	0.18	0.79	0.12	0.91
S12	0.16	0.16	0.36	0.71	0.63
S13	0.19	0.70	0.85	0.79	**1.53**
S14	0.25	0.67	0.62	**1.68**	0.97
S15	**1.95**	0.86	0.96	0.99	**1.40**
S16	0.29	0.22	0.18	0.42	0.45
S17	0.21	0.09	**1.59**	**1.29**	0.30
S18	0.17	0.98	**2.23**	**2.38**	**3.80**
S19	0.29	0.37	**1.02**	**1.70**	0.58
S20	0.61	0.11	0.29	**2.05**	**2.10**
S21	0.41	0.88	**5.29**	**4.15**	0.91
S22	0.92	0.25	0.74	**1.57**	0.42

**Amplitude ratios >1 indicate the main response at the second harmonic and are highlighted.**

For stimulations without foveal stray light suppression, two subjects showed the main response at the second harmonic for stimulations for stimulus eccentricity *r*_1_ = 0–1.6°. For *r*_2_ = 1.6–3.5°, one subject showed the main response at the second harmonic. Eleven subjects for *r*_3_ = 3.5–6.4° and twelve subjects for *r*_4_ = 6.4–10.9° showed main response at the second harmonic. For the stimulus eccentricity of *r*_5_ = 10.9–18°, 12 subjects had the main response at the second harmonic.

**FIGURE 6:**
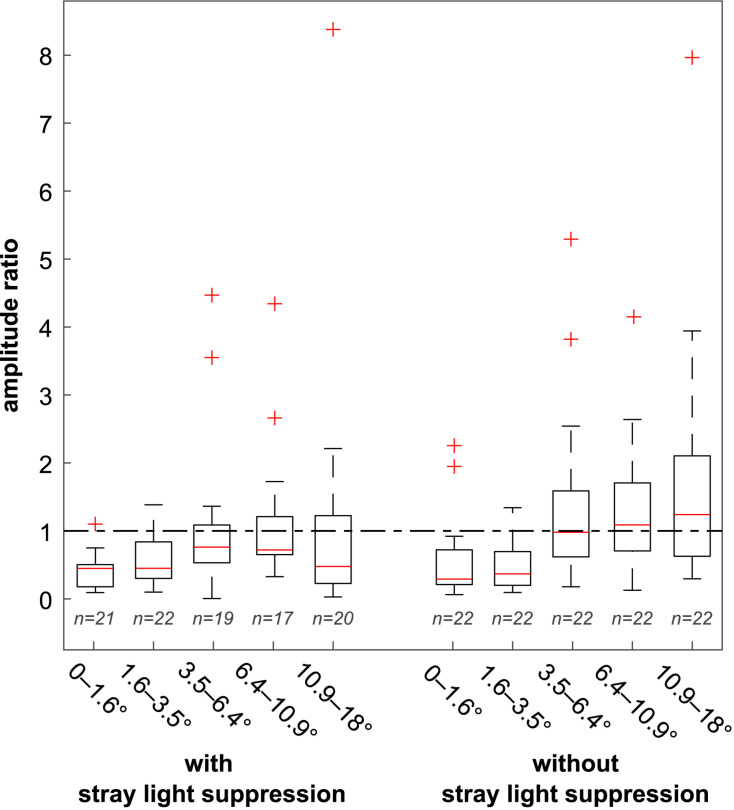
Distribution of the amplitude ratios for the stimulations with and without foveal stray light suppression. Medians are marked with a red line. The dash-dotted line marked the amplitude ratio 1. Amplitude ratios below 1 indicate a main response at the first harmonic, and a ratio above one indicates a main response at the second harmonic. For stimulations with foveal stray light suppression, the medians at all five eccentricities were below 1 (**M_0–1.6****°_** = 0.45, **M_1.6–3.5****°_** = 0.45, **M_3.5–6.4****°_** = 0.76, **M_6.4–10.9****°_** = 0.72, and **M_10.9–18****°_** = 0.48). For stimulations without foveal stray light suppression, the medians **M_0–1.6****°_** = 0.29, **M_1.6–3.5****°_** = 0.37, and **M_3.5–6.4****°_** = 0.98 were < 1 and **M_6.4–10.9****°_** = 1.08 and **M_10.9–18****°_** = 1.24 > 1, indicating a transition of the main response from the first harmonic to the second harmonic.

The distribution of the amplitude ratios is shown in Figure 6 in the form of boxplots. Medians are marked with a red line. The dash-dotted line marked the amplitude ratio 1. Amplitude ratios below 1 indicate a main response at the first harmonic, and a ratio above 1 indicates a main response at the second harmonic. The whiskers show the range of the data points to be within 1.5 times the interquartile range on the basis of the 75th percentile and 25th percentile, respectively. Outliers are defined as data points, larger and smaller than 1.5 times the interquartile distance on the basis of the 75th percentile and 25th percentile, respectively. For stimulations with foveal stray light suppression, the medians **M** at each eccentricity were <1 (**M**_**0**–**1.6**°_ = 0.45, **M**_**1.6**–**3.5**°_ = 0.45, **M**_**3.5**–**6.4**°_ = 0.76, **M**_**6.4**–**10.9**°_ = 0.72, and **M**_**10.9**–**18**°_ = 0.48), indicating that the main response occurred at the stimulation frequency. For stimulations without foveal stray light suppression, the medians **M**_**0**–**1.6**°_ = 0.29, **M**_**1.6**–**3.5**°_ = 0.37, and **M**_**3.5**–**6.4**°_ = 0.98 are <1 and for **M**_**6.4**–**10.9**°_ = 1.08 and **M**_**10.9**–**18**°_ = 1.24 > 1, indicating a transition of the main response from the first harmonic to the second harmonic.

**FIGURE 7:**
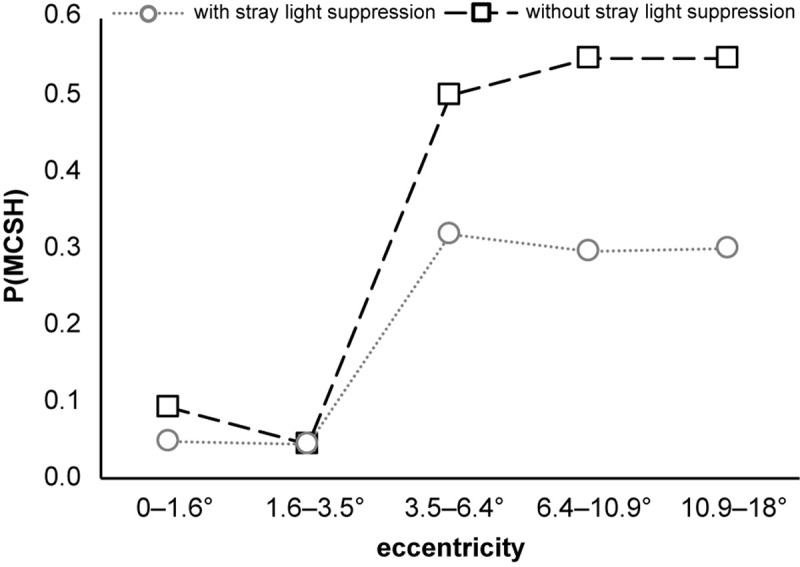
Probability of occurrence of the main response at the second harmonic **P(MCSH)** as a function of stimulus eccentricity. **P(MCSH)** increases abruptly for both stimulation conditions at stimulus eccentricity *r*_3_ = 3.5–6.4°. The sample size for the stimulations with foveal stray light suppression was 21 for *r*_1_ = 0–1.6°, 21 for *r*_2_ = 1.6–3.5°, 19 for *r*_3_ = 3.5–6.4°, 17 for *r*_4_ = 6.4–10.9°, and 20 for *r*_5_ = 10.9–18°. For the stimulations without foveal stray light suppression, the sample size for all eccentricities was 22.

The analysis of the probability of occurrence of the main response at the second harmonic **P(MCSH)** as a function of stimulus eccentricity for stimulations with and without foveal stray light suppression is shown in Figure 7. At the stimulus eccentricities *r*_1_ = 0–1.6° and *r*_2_ = 1.6–3.5°, **P(MCSH)** with and without stray light suppression is nearly in the same range [with stray light suppression: **P_0–1.6****°_(MCSH)** = 0.05, **P**_**1.6**–**3.5**°_**(MCSH)** = 0.05; without stray light suppression: **P**_**0**–**1.6**°_**(MCSH)** = 0.09, **P**_**1.6**–**3.5**°_**(MCSH)** = 0.05]. At stimulus eccentricity *r*_3_ = 3.5–6.4°, the **P(MCSH)** showed an abrupt increase for both types of stimulation, whereby the increase for stimulation without stray light suppression is stronger [**P**_**3.5**–**6.4**°_**(MCSH)** = 0.50] than that with stray light suppression [**P**_**3.5**–**6.4**°_**(MCSH)** = 0.32]. For eccentricities *r*_4_ = 6.4–10° and *r*_5_ = 10.9–18°, there is no noticeable increase in the probability of occurrence under either stimulation condition [with stray light suppression: **P**_**6.4** –1**0.9**°_**(MCSH)** = 0.29, **P**_**10.9**–**18**°_**(MCSH)** = 0.30; without stray light suppression: **P**_**6.4**–**10.9**°_**(MCSH)** = 0.55, **P**_**10.9**–**18**°_**(MCSH)** = 0.55].

## Discussion

The aim of this paper was to investigate the influence of stimulus design on the occurrence of the main response in ssVEPs. Two stimulus conditions were considered: first, stimulations with foveal stray light suppression and, second, stimulations without foveal stray light suppression. For analysis, the amplitude ratio of the amplitude of the frequency spectrum at the second harmonic to the first harmonic was calculated. The evaluation was based on the distribution of the amplitude ratio as well as the probability of occurrence of the main response at the second harmonic **P(MCSH)**. The medians **M** showed no transition of the main response to the second harmonic for the stimulations with foveal stray light suppression. For stimulations without stray light suppression, a transition of the main response to the second harmonic could be observed for **M**_**3.5**–**6.4**°_, **M**_**6.4**–**10.9**°_, and **M**_**10.9**–**18**°_. A comparable situation was found for the probability **P(MCSH)**. Stimulations without stray light suppression showed higher **P(MCSH)** compared to stimulations with foveal stray light suppression.

To achieve a comparable activated cortical area for stimulations at different stimulus eccentricity, the stimuli were scaled according to the cortical magnification. The frequency spectra of the 22 subjects show a significant difference for the amplitude values at first harmonic for the five stimulus eccentricities for stimulations with foveal stray light suppression (α = 0.05, after Bonferroni correction *p*^∗^ = 0.025, Friedman’s test: *p* = 0.000) and for stimulations without foveal stray light suppression (α = 0.05, after Bonferroni correction *p*^∗^ = 0.025, Friedman’s test: *p* = 0.002). One possible explanation for this is the calculation of the cortical area. The formula used to calculate the area contains empirically determined constants. There are many variants and adaptations to calculate the cortical magnification factor and the resulting cortical area (Rovamo and Virsu, 1979; Van Essen et al., 1984; Levi and Klein, 1985; Horton and Hoyt, 1991; Baseler et al., 1994; Slotnick et al., 2001; Duncan and Boynton, 2003). In addition, there is an intersubject variability due to the individual location and folding of the visual cortex (Stensaas et al., 1974; Hasnain et al., 1998; Heckenlively et al., 2006), which has an impact on the measured VEPs (Hood and Zhang, 2000; Vanegas et al., 2013). However, the influence of the variance of the amplitudes could be compensated by the normalization that ensued the calculation of the amplitude ratio.

A limitation of the study is due to the chosen stimulus design. For stimulations with foveal stray suppression, a background luminance of 30 cd/m^2^ was added to the stimulus. In the stimulations without stray light suppression, the background luminance was 0.5 cd/m^2^. The differences in background luminance may have had an effect on the adaptation states of the subjects.

Across all stimulations, an increase in the transition of the main response with increasing stimulus eccentricity was observed. The dependence of the amplitude ratio on stimulus eccentricity can be seen for stimulations with stray light suppression but no dominant transfer of the main response to the second harmonic occurred. However, a distinct increase with increasing stimulus eccentricity occurred for the stimulations without stray light suppression. At a stimulus eccentricity ≥*r*_3_ = 3.5–6.4°, about 50% of the subjects had a main response at the second harmonic. These results coincide with the study conducted by Solf et al. (2019b) using a comparable stimulus design with a peripheral stray light source (presented in the range of 5–10°). Fifty percentage of the tested persons also showed the main response at the second harmonic. For stimulations without stray light suppression, foveal stimulation by stray light occurred in addition to the peripheral stimulation. This leads to two possible effects. On the one hand, peripheral stimulations have shorter latencies in contrast to foveal stimulation (Yu and Brown, 1997; Kremláček et al., 2004). Furthermore, nasal presented stimuli have shorter latencies than temporal presented stimuli (Hood et al., 2000; Hood and Greenstein, 2003). On the other hand, brighter stimulations lead to a decrease in latency compared to darker stimulations. A saturation occurred from about 100 cd/m^2^ (Bach et al., 1985). In our stimulations, the stimuli had a luminance of 350 cd/m^2^. The resulting stray light had a luminance in the range of 2–3 cd/m^2^ which further increased the differences in latency. Assuming that latency differences result in signal components that cause second harmonic entrainment and resonance effects, this, in combination with the non-linearity of the visual system and the associated non-linear processing of the visual stimulations (Herrmann, 2001; Heinrich, 2010; Norcia et al., 2015; Labecki et al., 2016; Notbohm et al., 2016), may be an explanation for the weak increase in stimulations with stray light suppression as well as the stronger increase in stimulations without foveal stray light suppression. Investigating the dependence on stimulation contrast in foveal pattern-reversal stimulations, a slight increase of the amplitude ratio could be observed with increasing contrast. But, no transition of the main response to the second harmonic could be detected (Solf and Klee, 2019). However, as latency decreases with increasing stimulation contrast (Bobak et al., 1987; Jakobsson and Johansson, 1992; Tobimatsu et al., 1993; Kubová et al., 1995), this could also indicate the described effects.

As mentioned above, individual folding of the visual cortex had an impact on the recorded surface signals (Stensaas et al., 1974; Hasnain et al., 1998; Hood and Zhang, 2000; Heckenlively et al., 2006; Vanegas et al., 2013). The VEPs were recorded with an active electrode at Oz. Despite the standardization of the electrode position, the recorded VEP is evoked by the activities of different neuronal regions due to the individual shape and position of the visual cortex. Due to the intersubject neuroanatomical variability and the impact of the stimulus location (Clark et al., 1994), the response may not have been optimally covered by the electrode placement.

Looking at the distribution of the amplitudes as well as the **P(MCSH)**, a clear increase between the eccentricities *r*_2_ = 1.6–3.5° and *r*_3_ = 3.5–6.4°can be seen. Based on an extension of the fovea of about 5–5.5° depending on the definition of the fovea (Atchison and Smith, 2000; Hendrickson, 2009), a dependence of the harmonic components on the stimulus design can be derived. In foveal stimulation, the expression of the main response is hardly present in the second harmonic and is probably due to the individual variability. In the extrafoveal stimulation, a decrease in latency in peripheral stimulation (with stray light suppression) and additional non-linear processing of foveal and peripheral stimuli (without stray light suppression) seem to have an influence.

Thus, it can be concluded that the stimulus design has an influence on the expression of the main response. An increase in the stimulation eccentricity during extrafoveal stimulations leads to an increased transition of the main response to the second harmonic. The effect is enhanced by a simultaneous foveal stray light stimulation.

## Data Availability Statement

All datasets generated for this study are included in the article/Supplementary Material.

## Ethics Statement

The studies involving human participants were reviewed and approved by the Ethics Committee of the Faculty of Medicine of the Friedrich Schiller University Jena. The patients/participants provided their written informed consent to participate in this study.

## Author Contributions

BS: conceptualization, methodology, data acquisition and curation, data processing and analysis, manuscript drafting, and manuscript revision. SS and M-CB: conceptualization, methodology, and manuscript revision. SK: project administration and supervision, conceptualization, methodology, and manuscript revision. All authors contributed to the article and approved the submitted version.

## Conflict of Interest

The authors declare that the research was conducted in the absence of any commercial or financial relationships that could be construed as a potential conflict of interest.

## References

[B1] Almoqbel F. Leat S. J. Irving E. (2008). The technique, validity and clinical use of the sweep VEP. *Ophthalmic Physiol. Opt.* 28 393–403. 10.1111/j.1475-1313.2008.00591.x 18761477

[B2] Atchison D. Smith G. (2000). “Optics of the human eye,” in *Butterworth Heinemann*, (Amsterdam: Elsevier).

[B3] Bach M. Maurer J. Wolf M. (2008). Visual evoked potential-based acuity assessment in normal vision, artificially degraded vision, and in patients. *Br. J. Ophthalmol.* 92 396–403. 10.1136/bjo.2007.130245 18303162

[B4] Bach M. Meigen T. (1999). Do’s and don’ts in Fourier analysis of steady-state potentials. *Doc. Ophthalmol.* 99 69–82. 10947010 10.1023/a:1002648202420

[B5] Bach M. Waltenspiel S. Bühler B. Röver J. (1985). Sehbahndiagnostik mit simultaner Registrierung der retinalen und kortikalen Musterpotentiale. *Fortschr. Ophthalmol.* 82 398–401. 4054795

[B6] Baseler H. Sutter E. Klein S. Carney T. (1994). The topography of visual evoked response properties across the visual field. *Electroencephalogr. Clin. Neurophysiol.* 90 65–81. 10.1016/0013-4694(94)90114-7 7509275

[B7] Bobak P. Bodis-Wollner I. Guillory S. (1987). The effect of blur and contrast of VEP latency: comparison between check and sinusoidal grating patterns. *Electroencephalogr. Clin. Neurophysiol.* 68 247–255. 10.1016/0168-5597(87)90045-1 2439304

[B8] Clark V. P. Fan S. Hillyard S. A. (1994). Identification of early visual evoked potential generators by retinotopic and topographic analyses. *Hum. Brain Mapp.* 2 170–187. 10.1002/hbm.460020306

[B9] Cobb P. W. (1911). THE INFLUENCE OF ILLUMINATION OF THE EYE ON VISUAL ACUITY: I. Introductory and Historical. *Ame. J. Physiol. Leg. Content* 29 76–99. 10.1152/ajplegacy.1911.29.1.76

[B10] Duncan R. O. Boynton G. M. (2003). Cortical magnification within human primary visual cortex correlates with acuity thresholds. *Neuron* 38 659–671. 10.1016/s0896-6273(03)00265-4 12765616

[B11] Gulbinaite R. Roozendaal D. H. VanRullen R. (2019). Attention differentially modulates the amplitude of resonance frequencies in the visual cortex. *NeuroImage* 203:116146. 10.1016/j.neuroimage.2019.116146 31493535

[B12] Hasnain M. K. Fox P. T. Woldorff M. G. (1998). Intersubject variability of functional areas in the human visual cortex. *Hum. Brain Mapp.* 6 301–315. 10.1002/(sici)1097-0193(1998)6:4<301::aid-hbm8>3.0.co;2-7 9704267 PMC6873368

[B13] Heckenlively J. R. Arden G. B. Bach M. (2006). *Principles and Practice of Clinical Electrophysiology of Vision.* Cambridge, MA: MIT press.

[B14] Heinrich S. P. (2010). Some thoughts on the interpretation of steady-state evoked potentials. *Doc. Ophthalmol.* 120 205–214. 10.1007/s10633-010-9212-7 20101435

[B15] Heinrich S. P. Bock C. M. Bach M. (2016). Imitating the effect of amblyopia on VEP-based acuity estimates. *Doc. Ophthalmol.* 133 183–187. 10.1007/s10633-016-9565-7 27864655

[B16] Hendrickson A. (2009). “Fovea: primate,” in *Encyclopedia of the Neuroscience*, ed. Squire L. R. (Amsterdam: Elsevier).

[B17] Herrmann C. S. (2001). Human EEG responses to 1–100 Hz flicker: resonance phenomena in visual cortex and their potential correlation to cognitive phenomena. *Exp. Brain Res.* 137 346–353. 10.1007/s002210100682 11355381

[B18] Holladay L. (1926). The fundamentals of glare and visibility. *JOSA* 12 271–319.

[B19] Hood D. C. Greenstein V. C. (2003). Multifocal VEP and ganglion cell damage: applications and limitations for the study of glaucoma. *Prog. Retinal Eye Res.* 22 201–251. 10.1016/s1350-9462(02)00061-7 12604058

[B20] Hood D. C. Zhang X. (2000). Multifocal ERG and VEP responses and visual fields: comparing disease-related changes. *Doc. Ophthalmol.* 100 115–137. 11142742 10.1023/a:1002727602212

[B21] Hood D. C. Zhang X. Greenstein V. C. Kangovi S. Odel J. G. Liebmann J. M. (2000). An interocular comparison of the multifocal VEP: a possible technique for detecting local damage to the optic nerve. *Invest. Ophthalmol. Vis. Sci.* 41 1580–1587. 10798679

[B22] Horton J. C. Hoyt W. F. (1991). The representation of the visual field in human striate cortex: a revision of the classic Holmes map. *Arch. Ophthalmol.* 109 816–824. 2043069 10.1001/archopht.1991.01080060080030

[B23] Jakobsson P. Johansson B. (1992). The effect of spatial frequency and contrast on the latency in the visual evoked potential. *Doc. Ophthalmol.* 79 187–194. 10.1007/bf00156577 1591972

[B24] Johansson B. Jakobsson P. (2000). Fourier analysis of steady-state visual evoked potentials in subjects with normal and defective stereo vision. *Doc. Ophthalmol.* 101 233–246. 11291952 10.1023/a:1002876804178

[B25] Kremláček J. Kuba M. Chlubnová J. Kubová Z. (2004). Effect of stimulus localisation on motion-onset VEP. *Vis. Res.* 44 2989–3000. 10.1016/j.visres.2004.07.002 15474572

[B26] Kubová Z. Kuba M. Spekreijse H. Blakemore C. (1995). Contrast dependence of motion-onset and pattern-reversal evoked potentials. *Vis. Res.* 35 197–205. 10.1016/0042-6989(94)00138-c 7839616

[B27] Labecki M. Kus R. Brzozowska A. Stacewicz T. Bhattacharya B. S. Suffczynski P. (2016). Nonlinear origin of SSVEP spectra—a combined experimental and modeling study. *Front. Comput. Neurosci.* 10:129. 10.3389/fncom.2016.00129 28082888 PMC5187367

[B28] Levi D. M. Klein S. A. (1985). Vernier acuity, crowding and amblyopia. *Vis. Res.* 25 979–991. 10.1016/0042-6989(85)90208-1 4049747

[B29] Meigen T. Bach M. (1999). On the statistical significance of electrophysiological steady-state responses. *Doc. Ophthalmol.* 98 207–232. 10945442 10.1023/a:1002097208337

[B30] Norcia A. M. Appelbaum L. G. Ales J. M. Cottereau B. R. Rossion B. (2015). The steady-state visual evoked potential in vision research: a review. *J. Vis.* 15:4 10.1167/15.6.4 PMC4581566 26024451

[B31] Norcia A. M. Tyler C. W. (1985). Spatial frequency sweep VEP: visual acuity during the first year of life. *Vis. Res.* 25 1399–1408. 10.1016/0042-6989(85)90217-2 4090273

[B32] Norcia A. M. Tyler C. W. Hamer R. D. Wesemann W. (1989). Measurement of spatial contrast sensitivity with the swept contrast VEP. *Vis. Res.* 29 627–637. 10.1016/0042-6989(89)90048-5 2603399

[B33] Notbohm A. Kurths J. Herrmann C. S. (2016). Modification of brain oscillations via rhythmic light stimulation provides evidence for entrainment but not for superposition of event-related responses. *Front. Hum. Neurosci.* 10:10. 10.3389/fnhum.2016.00010 26869898 PMC4737907

[B34] Odom J. V. Bach M. Brigell M. Holder G. E. McCulloch D. L. Mizota A. (2016). ISCEV standard for clinical visual evoked potentials:(2016 update). *Doc. Ophthalmol.* 133 1–9. 10.1007/s10633-016-9553-y 27443562

[B35] Regan D. (1966). Some characteristics of average steady-state and transient responses evoked by modulated light. *Electroencephalogr. Clin. Neurophysiol.* 20 238–248. 10.1016/0013-4694(66)90088-5 4160391

[B36] Regan D. (1973). Rapid objective refraction using evoked brain potentials. *Invest. Ophthalmol. Vis. Sci.* 12 669–679. 4742063

[B37] Rovamo J. Virsu V. (1979). An estimation and application of the human cortical magnification factor. *Exp. Brain Res.* 37 495–510. 520439 10.1007/BF00236819

[B38] Salchow C. Strohmeier D. Klee S. Jannek D. Schiecke K. Witte H. (2016). Rod driven frequency entrainment and resonance phenomena. *Front. Hum. Neurosci.* 10:413. 10.3389/fnhum.2016.00413 27588002 PMC4989477

[B39] Slotnick S. D. Klein S. A. Carney T. Sutter E. E. (2001). Electrophysiological estimate of human cortical magnification. *Clin. Neurophysiol.* 112 1349–1356. 10.1016/s1388-2457(01)00561-2 11516748

[B40] Solf B. Klee S. (2019). The effect of stimulus contrast on the harmonic components of steady state visual evoked potentials. *Acta Ophthalmol.* 97.

[B41] Solf B. Schramm S. Klee S. (2019a). The effect of stray light on the electrophysiological measurement of the contrast threshold. *Biomed. Eng. Biomed. Tech.* 64 (Suppl. 2):177.

[B42] Stensaas S. S. Eddington D. K. Dobelle W. H. (1974). The topography and variability of the primary visual cortex in man. *J. Neurosurg.* 40 747–755. 10.3171/jns.1974.40.6.0747 4826600

[B43] Solf B. Schramm S. Link D. Klee S. (2019b). Objective measurement of forward-scattered light in the human eye: an electrophysiological approach. *PLoS One* 14:e0214850. 10.1371/journal.pone.0214850 30947303 PMC6448911

[B44] Stiles W. S. (1929). The effect of glare on the brightness difference threshold. *Proc. R. Soc. Lon. Ser. B* 104 322–351. 10.1098/rspb.1929.0012

[B45] Tobimatsu S. Kurita-Tashima S. Nakayama-Hiromatsu M. Akazawa K. Kato M. (1993). Age-related changes in pattern visual evoked potentials: differential effects of luminance, contrast and check size. *Electroencephalogr. Clin. Neurophysiol.* 88 12–19. 10.1016/0168-5597(93)90023-i 7681386

[B46] Tyler C. W. Apkarian P. Levi D. M. Nakayama K. (1979). Rapid assessment of visual function: an electronic sweep technique for the pattern visual evoked potential. *Invest. Ophthalmol. Vis. Sci.* 18 703–713. 447469

[B47] van den Berg T. J. (1995). Analysis of intraocular straylight, especially in relation to age. *Opt. Vis. Sci.* 72 52–59. 10.1097/00006324-199502000-00003 7753528

[B48] Van Essen D. C. Newsome W. T. Maunsell J. H. (1984). The visual field representation in striate cortex of the macaque monkey: asymmetries, anisotropies, and individual variability. *Vis. Res.* 24 429–448. 10.1016/0042-6989(84)90041-5 6740964

[B49] Vanegas M. I. Blangero A. Kelly S. P. (2013). Exploiting individual primary visual cortex geometry to boost steady state visual evoked potentials. *J. Neural Eng.* 10:036003 10.1088/1741-2560/10/3/036003 PMC3660541 23548662

[B50] Yu M. Z. Brown B. (1997). Variation of topographic visually evoked potentials across the visual field. *Ophthalmic Physiol. Opt.* 17 25–31. 10.1111/j.1475-1313.1997.tb00520.x 9135809

