# Corrigendum: The Influence of the Stimulus Design on the Harmonic Components of the Steady-State Visual Evoked Potential

**DOI:** 10.3389/fnhum.2020.644304

**Published:** 2021-01-26

**Authors:** Benjamin Solf, Stefan Schramm, Maren-Christina Blum, Sascha Klee

**Affiliations:** Institute for Biomedical Engineering and Informatics, Technische Universität Ilmenau, Ilmenau, Germany

**Keywords:** steady-state visual evoked potentials, ssVEP, flicker stimulation, harmonic components, stimulus eccentricity, ocular stray light

In the original article, there was an inaccurate statement. In the introduction, we cited studies analyzing the effect of stimulation parameters on the harmonic components of ssVEP. Thereby, we made the statement that the studies did not analyze the occurrence of the main response at the harmonic components. After a cited author pointed out that the responses at higher harmonic components were analyzed in their study, we revised our statement.

A correction has been made to *Introduction, Paragraph 4*. The corrected paragraph is shown below:

The effect of the stimulation parameters on the harmonic components of ssVEPs was analyzed in some studies. For example, Johansson and Jakobsson (2000) found significant higher amplitudes at high temporal frequencies in normal subjects than in stereo-blind subjects. Gulbinaite et al. (2019) analyzed the effect of attention on the amplitudes of the harmonic components for stimulation frequencies within the range of 3–80 Hz and found an opposite effect of attention on the individual resonance frequencies in the alpha and gamma band.

The authors apologize for this error and state that this does not change the scientific conclusions of the article in any way. The original article has been updated.
