# Supplementary material for: The Influence of the Stimulus Design on the Harmonic Components of the Steady-State Visual Evoked Potential
Source: Front Hum Neurosci. 2020 Sep 9;14:343. doi: 10.3389/fnhum.2020.00343 (PMC7509136; doi:10.3389/fnhum.2020.00343)
Supplement: Supplementary file 1 [file Table_1.docx]

Supplementary Material

**Supplementary Table 1**: P values for the significance of the amplitudes of the first and second harmonics for stimulations with stray light suppression. Due to multiple testing, the significance level of α = 0.05 was set to an adjusted significance value of p* = 0.025. Nonsignificant responses are highlighted.

| **p values** | | | | | | | | | | | |
| --- | --- | --- | --- | --- | --- | --- | --- | --- | --- | --- | --- |
|  | | 0–1.6° | | 1.6–3.5° | | 3.5–6.4° | | 6.4–10.9° | | 10.9–18° | |
| **Subject** | 7.5 Hz | | 15 Hz | 7.5 Hz | 15 Hz | 7.5 Hz | 15 Hz | 7.5 Hz | 15 Hz | 7.5 Hz | 15 Hz |
| S01 | 0.001 | | **0.039** | 0.002 | 0.000 | 0.008 | 0.002 | 0.004 | 0.011 | 0.012 | **0.588** |
| S02 | 0.000 | | **0.321** | 0.000 | 0.000 | 0.000 | 0.002 | **0.200** | **0.255** | **0.326** | **0.402** |
| S03 | 0.000 | | 0.000 | 0.002 | **0.618** | **0.141** | 0.020 | 0.003 | 0.011 | 0.004 | 0.003 |
| S04 | 0.000 | | 0.013 | 0.002 | 0.003 | 0.000 | 0.018 | 0.007 | 0.020 | 0.007 | 0.000 |
| S05 | 0.000 | | 0.007 | 0.001 | 0.001 | 0.001 | 0.001 | 0.000 | 0.005 | 0.000 | **0.091** |
| S06 | 0.000 | | 0.001 | 0.002 | **0.245** | **0.126** | **0.385** | **0.103** | **0.059** | **0.098** | **0.069** |
| S07 | 0.000 | | 0.000 | 0.000 | 0.000 | 0.000 | 0.000 | 0.000 | 0.000 | 0.004 | 0.000 |
| S08 | 0.001 | | 0.007 | 0.005 | 0.001 | 0.013 | 0.005 | **0.554** | **0.047** | **0.032** | 0.005 |
| S09 | 0.000 | | 0.005 | 0.006 | 0.005 | **0.092** | 0.005 | 0.000 | 0.000 | 0.001 | 0.009 |
| S10 | 0.002 | | 0.001 | 0.000 | 0.005 | 0.007 | **1.000** | **0.074** | **0.044** | **0.037** | 0.010 |
| S11 | 0.000 | | 0.005 | 0.001 | 0.000 | 0.001 | 0.000 | 0.001 | **0.048** | 0.001 | 0.011 |
| S12 | 0.000 | | 0.000 | 0.001 | 0.001 | 0.003 | 0.001 | 0.008 | 0.006 | 0.023 | 0.004 |
| S13 | 0.000 | | 0.000 | 0.001 | 0.000 | **0.056** | 0.001 | 0.010 | 0.000 | 0.000 | 0.002 |
| S14 | 0.000 | | 0.000 | 0.009 | 0.007 | 0.000 | 0.003 | 0.000 | 0.000 | 0.018 | 0.006 |
| S15 | 0.000 | | **0.141** | 0.002 | 0.014 | **0.035** | 0.015 | **0.255** | 0.001 | **0.801** | 0.000 |
| S16 | 0.005 | | 0.009 | 0.004 | **0.041** | **0.251** | **0.438** | **0.680** | 0.020 | **0.070** | 0.001 |
| S17 | 0.000 | | 0.002 | 0.001 | **0.033** | **0.356** | 0.010 | **0.074** | **0.112** | 0.006 | 0.004 |
| S18 | 0.000 | | 0.000 | 0.005 | 0.001 | **0.069** | **0.041** | 0.009 | 0.013 | **0.153** | 0.013 |
| S19 | 0.000 | | 0.000 | 0.003 | 0.005 | **0.108** | 0.011 | 0.004 | 0.016 | 0.001 | 0.004 |
| S20 | 0.003 | | 0.001 | 0.000 | 0.000 | 0.003 | 0.000 | 0.013 | 0.007 | **0.026** | 0.001 |
| S21 | 0.000 | | 0.002 | 0.000 | **0.029** | 0.001 | 0.006 | 0.018 | 0.001 | 0.005 | 0.003 |
| S22 | **0.087** | | **0.029** | **0.037** | 0.001 | 0.011 | 0.022 | 0.001 | 0.000 | 0.009 | **0.823** |

**Supplementary Table 2**: P values for the significance of the amplitudes of the first and second harmonics for stimulations without stray light suppression. Due to multiple testing, the significance level of α = 0.05 was set to an adjusted significance value of p* = 0.025. Nonsignificant responses are highlighted.

| **p values** | | | | | | | | | | | |
| --- | --- | --- | --- | --- | --- | --- | --- | --- | --- | --- | --- |
|  | | 0–1.6° | | 1.6–3.5° | | 3.5–6.4° | | 6.4–10.9° | | 10.9–18° | |
| **Subject** | 7.5 Hz | | 15 Hz | 7.5 Hz | 15 Hz | 7.5 Hz | 15 Hz | 7.5 Hz | 15 Hz | 7.5 Hz | 15 Hz |
| S01 | 0.000 | | 0.000 | 0.000 | 0.001 | 0.001 | 0.000 | 0.000 | 0.001 | 0.000 | 0.000 |
| S02 | 0.003 | | 0.007 | 0.000 | 0.000 | 0.009 | 0.000 | 0.001 | 0.000 | 0.002 | 0.000 |
| S03 | 0.001 | | 0.000 | 0.000 | **0.040** | **0.043** | 0.000 | 0.004 | 0.000 | **0.026** | 0.000 |
| S04 | 0.000 | | **0.068** | 0.000 | 0.002 | 0.000 | 0.001 | 0.001 | 0.000 | 0.003 | 0.004 |
| S05 | 0.000 | | 0.000 | 0.001 | 0.000 | 0.000 | 0.000 | 0.000 | 0.000 | 0.000 | 0.000 |
| S06 | 0.000 | | 0.002 | 0.000 | 0.000 | 0.003 | 0.000 | 0.000 | 0.001 | 0.000 | 0.000 |
| S07 | 0.000 | | 0.000 | 0.001 | 0.007 | 0.001 | 0.004 | 0.000 | 0.000 | 0.000 | 0.001 |
| S08 | **0.672** | | 0.004 | 0.000 | 0.000 | **0.061** | 0.000 | 0.002 | 0.001 | 0.006 | 0.001 |
| S09 | 0.000 | | 0.002 | 0.002 | 0.000 | 0.014 | 0.000 | 0.000 | 0.000 | **0.116** | 0.000 |
| S10 | 0.000 | | 0.021 | 0.002 | 0.007 | **0.041** | 0.002 | 0.010 | 0.001 | 0.001 | 0.000 |
| S11 | 0.000 | | 0.011 | 0.000 | 0.004 | 0.004 | 0.000 | 0.005 | **0.128** | 0.001 | 0.000 |
| S12 | 0.001 | | 0.014 | 0.000 | 0.004 | 0.000 | 0.003 | 0.002 | 0.000 | 0.000 | 0.000 |
| S13 | 0.001 | | 0.024 | 0.001 | 0.000 | 0.007 | 0.000 | **0.143** | 0.003 | 0.024 | 0.004 |
| S14 | 0.000 | | 0.000 | 0.000 | 0.000 | 0.000 | 0.001 | 0.002 | 0.000 | 0.004 | 0.000 |
| S15 | **0.036** | | 0.000 | 0.009 | 0.000 | **0.051** | 0.000 | 0.011 | 0.000 | 0.004 | 0.000 |
| S16 | **0.058** | | 0.003 | 0.003 | **0.040** | 0.008 | **0.163** | 0.006 | 0.000 | **0.110** | 0.013 |
| S17 | 0.000 | | 0.024 | 0.000 | **0.223** | **0.035** | 0.000 | **0.055** | 0.000 | 0.000 | 0.007 |
| S18 | 0.000 | | 0.001 | 0.002 | 0.000 | 0.001 | 0.000 | 0.001 | 0.000 | **0.357** | 0.004 |
| S19 | 0.001 | | 0.008 | 0.001 | 0.001 | 0.003 | 0.000 | 0.002 | 0.000 | 0.000 | 0.000 |
| S20 | 0.003 | | 0.004 | 0.001 | 0.021 | 0.000 | 0.000 | 0.000 | 0.000 | 0.000 | 0.000 |
| S21 | 0.000 | | 0.001 | 0.000 | 0.000 | 0.003 | 0.000 | 0.001 | 0.000 | 0.000 | 0.000 |
| S22 | 0.002 | | 0.000 | 0.000 | 0.015 | 0.000 | 0.001 | 0.003 | 0.001 | 0.000 | 0.000 |
